# Surgical Stress Delays Prostate Involution in Mice

**DOI:** 10.1371/journal.pone.0078175

**Published:** 2013-11-06

**Authors:** Sazzad Hassan, Yelena Karpova, Anabel Flores, Ralph D’Agostino, George Kulik

**Affiliations:** 1 Department of Cancer Biology, Wake Forest School of Medicine, Winston-Salem, North Carolina, United States of America; 2 Comprehensive Cancer Center, Wake Forest School of Medicine, Winston-Salem, North Carolina, United States of America; 3 College of Science, Alfaisal University, Riyadh, Saudi Arabia; University of Missouri-Columbia, United States of America

## Abstract

Androgens control growth of prostate epithelial cells and androgen deprivation induces apoptosis, leading to prostate involution. We investigated the effects of surgical stress on prostate involution induced by androgen ablation and determined the underlying mechanisms. Androgen ablation in mice was induced by surgical castration and administration of the anti-androgenic drugs bicalutamide and MDV3100. Surgical stress was induced by sham castration under isoflurane anesthesia. Surgical stress delayed apoptosis and prostate involution induced by anti-androgenic drugs. These effects of stress were prevented by administering the selective beta2-adrenoreceptor antagonist ICI118,551 and were also blocked in BAD^3SA/WT^ mice expressing phosphorylation-deficient mutant BAD3SA. These results indicate that apoptosis and prostate involution in response to androgen ablation therapy could be delayed by surgical stress via the beta2-adrenoreceptor/BAD signaling pathway. Thus, surgery could interfere with androgen ablation therapy, whereas administration of beta2-adrenoreceptor antagonists may enhance its efficacy.

## Introduction

Prostate cancer (PC) is a common malignancy and is second only to lung cancer as a cause of death in men. Anti-androgen therapy is effective in the early stages of PC. Unfortunately, within 18 months of androgen withdrawal, PC progresses to androgen-independent disease for which no curative treatments are yet available [1]. This relapse with progressive growth and metastasis of PC cancers depends on increased sensitivity of androgen receptors (AR) to castrate levels of androgens, androgen-independent activation of AR, or activation of signaling mechanisms that promote growth and survival of prostate cancer cells via AR-independent signaling pathways [2]. Therefore, information about the extrinsic and intrinsic factors decreasing the sensitivity of prostate epithelial cells to therapies that target AR signaling would be useful for improving efficacy of androgen ablation therapies.

Substantial numbers of patients receiving androgen-ablation therapy with androgen receptor antagonists may need subsequent surgery to treat complications of prostate cancer or for unrelated reasons [3]–[5]. Surgery has been reported to facilitate growth of several types of tumors, including colon and ovarian cancer [6]–[8]. However, effects of surgical stress on apoptosis and prostate involution after androgen ablation therapy have not been studied.

We examined whether behavioral and surgical stress can influence prostate involution induced by androgen ablation in mice and investigated mechanisms of surgical stress signaling. Surgical stress inhibited apoptosis and delayed prostate involution induced by androgen-ablation therapies in mouse prostate glands. Activation of the epinephrine/beta2 adrenergic receptor/PKA and BAD signaling pathway prevented apoptosis induced by anti-androgenic drugs in androgen-dependent prostate cells. These results define a mechanism by which surgery could interfere with androgen ablation therapy. Future studies that compare efficacies of androgen ablation therapies in patients who underwent surgeries are needed to inform whether targeting β2-adrenoreceptor signaling in prostate cancer patients is justified.

## Methods

### Immobilization Stress, Bicalutamide and ICI118,551 in Mice

12-week-old C57BL/6 mice were subjected to recurrent 1-hr immobilization stress at 12-hr intervals for 1–3 consecutive days [9]. Bicalutamide (50 mg/kg) was injected subcutaneously once a day. To investigate the role of the β_2_-adrenergic receptor in stress effects, the β-adrenergic receptor antagonist ICI118551 (25 µM, 30 µl) was given intraperitoneally 30 minutes before immobilization stress. At 24 hours after the last injection, blood and prostate tissues were collected and processed [9].

### Surgical Stress in Mice

12-week-old mice were subjected to surgical stress by castration or sham operation. Briefly, after the initiation of general isoflurane anesthesia, the testes were gently pushed into the scrotum and a 0.5 cm incision was made. The spermatic vessels were tied with 4.0 silk sutures and the testes were removed. The incision was then closed with 4.0 silk sutures. In sham-operated mice, the skin of the scrotum was incised to draw out and back the testes and closed with sutures only. Bicalutamide and ICI118551 were then given as described above.

### Adrenaline ELISAs

Plasma adrenaline concentrations were measured by ELISA (Adrenaline EIA; LDN, Nordhorn, Germany) [9].

### Antibodies

Antibodies were from the following sources: phospho-specific BAD (Ser112), phospho-CREB (Ser133), and cleaved caspase 3 from Cell Signaling Technology (Beverly, MA); mouse monoclonal antibody to β-actin from Sigma (St. Louis, MO); goat polyclonal antibody to cleaved PARP from R&D (R&D Systems, Minneapolis, MN); and secondary horseradish peroxidase-conjugated antibodies used for Western blots from Amersham Biosciences (Piscataway, NJ). Secondary goat anti-mouse IRDye 680 and goat-anti-rabbit IRDye 800 were both purchased from Li-Cor Biosciences. Protein bands were quantified using ImageJ software (National Institutes of Health, Bethesda, MD, USA).

### Western Blot Analysis

Tissue or cell lysates with equal amounts of total protein were electrophoresed and transferred to nitrocellulose membranes for immunoblotting with appropriate antibodies. The membranes were incubated with primary antibodies overnight at 4°C followed by 1 hour of incubation at room temperature with the secondary antibodies. Proteins were visualized using an ECL chemiluminescence detection system (Amersham) and Odyssey CLx Infrared Imaging System (Li-Cor Biosciences, Lincoln, NE, USA) according to the manufacturer’s instructions. After staining, blots were stripped and reprobed with different antibodies for comparison and normalization.

### Immunohistochemistry and Immunofluorescence

Antibody staining for cleaved caspase-3 (catalog 9661; Cell Signaling Technology) was performed on histological sections of formalin-fixed mice prostate lobes as described earlier [9]. Cleaved caspase 3 and TUNEL immunofluorescence was done as described previously [10].

### Statistical Analysis

Student’s t-test analysis (two-tailed distribution; two-sample unequal variance) was performed using Excel software. Power calculations were conducted using SAS software. For analysis of castration-induced prostate involution – based on the estimated pooled standard deviation observed in the 2BC and 2BS groups of 6.84 there was 80% power to detect a difference between these groups equal to 13.0 mg/25 g using a two group t-test with alpha = 0.05 (2-sided test) For experiments with analysis of prostate involution induced by bicalutamide and MDV3100 based on the estimated pooled standard deviation observed in Groups 2 (bicalutamide), 3 (sham castration + bicalutamide), and 5 (castration+bicalutamide + ICI) of 5.54, there was 80% power to detect a difference equal to 14.9 mg/25 g (for comparing group 3 vs group 5) or 13.6 mg/25 g (when comparing group 2 vs 3) using a two group t-test with alpha = 0.05 (2-sided test).

### Study Approval

All animal studies were conducted according to a protocol approved by the Institutional Animal Care and Use Committee of Wake Forest School of Medicine and conformed to NIH guidelines.

## Results

### Behavioral Stress Failed to Delay Surgical Castration-induced Involution and Apoptosis in Mice Prostates

**Figure 1:**
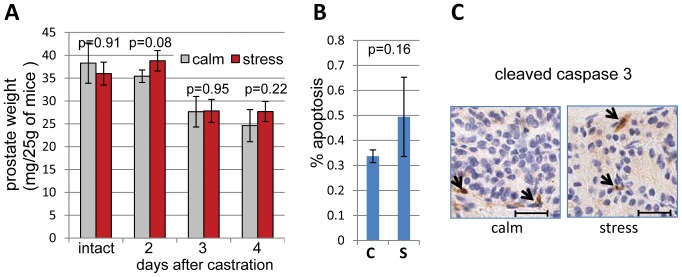
Behavioral stress fails to delay surgical castration-induced involution and apoptosis in mice prostates. **A)** Castration-induced prostate involution. Mice were either left intact (n = 4) or surgically castrated and subjected to immobilization stress twice a day with 12 h intervals (stress) or not subjected to stress (calm). At 2, 3 or 4 days post-castration (n = 3 for both calm and stress), prostates were excised, dissected, and weighted. Prostate weight decreased significantly by day 3 after castration (p = 0.01). There were no significant differences in prostate weights between stressed and calm groups. **B)** Stress does not decrease apoptosis in castrated prostates. Apoptosis (expressed as percent cleaved caspase-3 positive cells) was determined relative to the total number of glandular epithelial cells in whole sections of dorsolateral prostate glands excised 3 days after castration in stressed (S) and calm (C) groups of mice (n = 3 in each group). **C)** Representative images of cleaved caspase-3 IHC-stained sections from dorsolateral segments of the prostate from intact (calm) or stressed (stress) mice. Arrows point at caspase 3 positive cells. Scale bars: 50 µm.

We tested whether immobilization stress would delay the involution of prostate gland induced by surgical castration and observed no effects of immobilization stress in castrated mice. No significant increases in prostate weights or decreases in apoptosis were detected in mice subjected to repeated immobilization stress after castration, compared to mice not subjected to stress (Fig. 1). These data contrast with the results of our recent experiments that showed decreased apoptosis induced by bicalutamide and increased prostate weight in *Hi-Myc* mice subjected to immobilization stress [9].

Our initial hypothesis was that unlike apoptosis induced by bicalutamide in *Hi-Myc* mice, apoptosis induced by androgen ablation in C57BL/6 wild type mice is not susceptible to inhibition by stress signaling. To test this hypothesis, we examined effects of psychoemotional stress on prostate involution induced by bicalutamide in C57BL/6 mice.

### Behavioral Stress Delays Androgen Ablation-induced Prostate Involution and Apoptosis via β2-AR and BAD Phosphorylation

**Figure 2:**
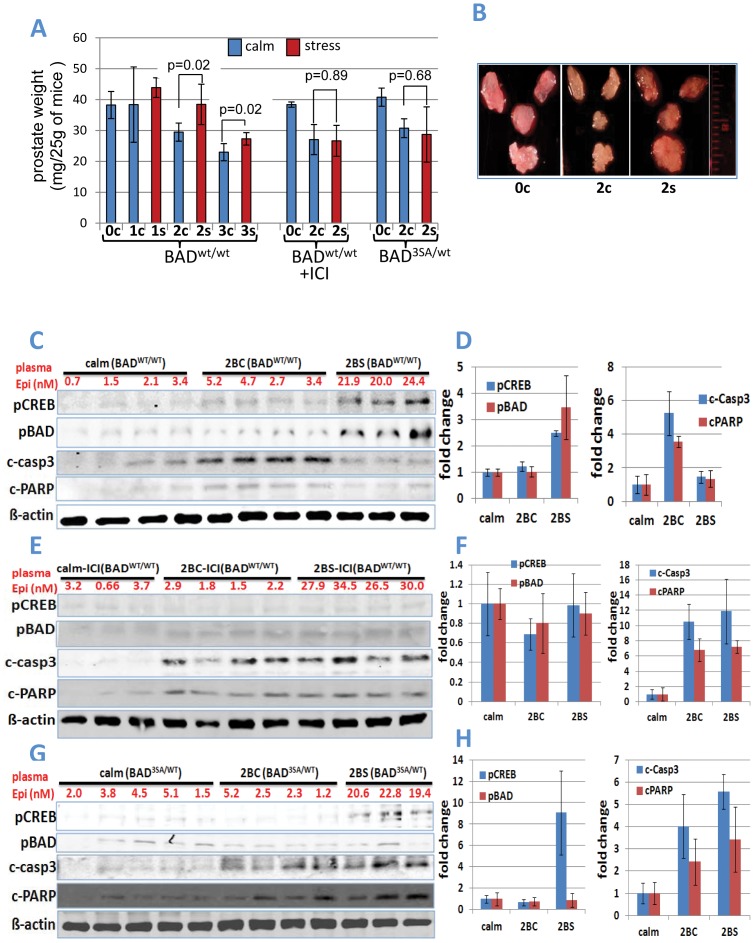
Behavioral stress delays androgen ablation-induced prostate involution and apoptosis via β2-AR and BAD phosphorylation. **A)** Stress attenuates bicalutamide-induced decrease in prostate weight of mice via ADRB2/BAD signaling. S = stressed; C = calm. Intact calm mice (0c), numbers under the columns indicate days of bicalutamide treatment; BAD^wt/wt^ mice with wild type BAD; BAD^wt/wt^ +ICI, mice treated with ICI118,551; BAD^3SA/WT^, mice with knockin on phosphorylation-deficient BAD3SA mutant (n = 3 to 5 in each group). **B)** Microphotographs of dissected prostate glands from intact wild-type (**0c**), calm mice after 2 injections of bicalutamide (**2c**), and stressed mice after 2 injections of bicalutamide (**2s**). (**C–H**) Western blot and densitometric analysis of Western blots of proteins extracted from of anterior prostate glands of wild-type mice (BAD^WT/WT^) (C, D); wild-type mice treated with ICI118,551 (ICI, BAD^WT/WT^) (E, F); and transgenic mice that express phosphorylation-deficient mutant BAD (BAD^3SA/WT^) (G, H). (**C, E, G**) Western blots conducted with antibodies to pSer133CREB (pCREB), pSer112BAD (pBAD), cleaved caspase 3 (c-casp3), cleaved PARP (cPARP) and β-actin. 3–4 representative samples for each treatment group are shown. Numbers over the Western blots show plasma epinephrine levels. Calm(BAD^WT/WT^), mice with wild type BAD that were not stressed; 2BC(BAD^WT/WT^), mice with wild type BAD that were given bicalutamide for 2 days and not stressed; 2BS(BAD^WT/WT^), mice with wild type BAD that were given bicalutamide for 2 days and stressed; calm-ICI(BAD^WT/WT^), mice with wild type BAD that were given ICI118,551 for 2days and not stressed; 2BC-ICI(BAD^WT/WT^), mice with wild type BAD that were given bicalutamide and ICI118,551 for 2 days and not stressed; 2BS-ICI(BAD^WT/WT^), mice with wild type BAD that were given bicalutamide and ICI118,551 for 2 days and stressed; calm(BAD^3SA/WT^), mice with phosphorylation deficient mutated BAD that were not stressed; 2BC(BAD^3SA/WT^), mice with phosphorylation deficient mutated BAD that were given bicalutamide for 2 days and not stressed; 2BS(BAD^3SA/WT^), mice with phosphorylation deficient mutated BAD that were given bicalutamide for 2 days and stressed. (**D, F, H**) BAD and CREB phosphorylation was increased in stressed mice and reduced cleavage of caspase 3 and PARP was reduced in stressed mice treated with bicalutamide (*p<0.03). These effects were completely eliminated in mice injected with ICI118,551 (ICI) and in BAD3SA (BAD+/−) knock-in mice. Each experimental group contained at least 5 mice. Error bars represent SD from the average.

Bicalutamide treatment induced apoptosis in normal prostate epithelial cells and involution of prostate glands in mice. In mice subjected to immobilization stress, prostate involution was delayed. Prostate weights were significantly higher in stressed mice on the second and third days of bicalutamide treatment, but larger differences were seen on day 2 (Fig. 2 A, B). Consistent with increased apoptosis, cleaved caspase 3 and cleaved PARP were detected in prostates of “calm” mice that received bicalutamide for 2 days. In prostates of mice subjected to immobilization stress, S112BAD and S133CREB phosphorylation increased, whereas caspase 3 and PARP cleavage was prevented (Fig. 2 C, D).

To test the role of epinephrine/beta2AR/BAD signaling axis in the effects of stress on prostate weight, we compared the effects of stress on prostate involution after administration of the ADRB2 selective antagonist ICI118,551 and in BAD3SA knockin mice. ICI118,551 completely blocked the effects of immobilization stress on prostate weight and apoptosis (Fig. 2A,E,F). Likewise, effects of stress on prostate weight and apoptosis were eliminated in the prostates of transgenic BAD^WT/3SA^ mice with knock-in of phosphorylation-deficient mutant BAD3SA (Fig. 2A,G,H). ICI118,551 inhibited both CREB and BAD phosphorylation in prostates of stressed mice (Fig. 2EF), although in prostates of BAD^WT/3SA^ mice, CREB was still phosphorylated (Fig. 2 G, H). Despite increased CREB phosphorylation, stress did not decrease apoptosis or increase prostate weights in BAD^WT/3SA^ mice (Fig. 2 A, G, H). Thus, BAD phosphorylation is a regulatory target for apoptosis downstream of PKA. Taken together, these results suggest that in normal prostates, immobilization stress inhibits apoptosis and prostate involution via the epinephrine/ADRB2/BAD signaling pathway, as we reported earlier in *Hi-Myc* mice.

### Surgical Stress Increases Adrenaline Levels

**Figure 3:**
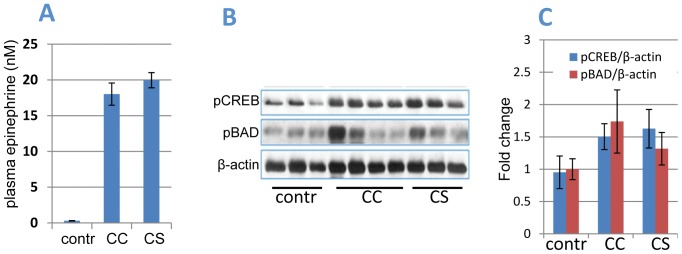
Surgical castration increases epinephrine and activates the PKA/BAD signaling pathway. **A)** Epinephrine levels in plasma of castrated mice either left intact or subjected to immobilization stress for 1 h. Contr = intact mice; CC = castrated and not stressed (calm); CS = castrated and stressed. **B, C)** Increased BAD and CREB phosphorylation in prostates of castrated mice. (B) Western blot and (C) densitometric analysis of Western blots of proteins extracted from anterior prostate glands of same mice as in (A). At least 3 mice were used for each data point. Error bars represent SD from the average. Contr = intact mice; CC = castrated and not stressed (calm); CS = castrated and stressed.

Next, we hypothesized that surgically castrated mice have a subverted response to immobilization stress. To test this hypothesis, we measured epinephrine levels in castrated mice left calm or subjected to immobilization stress. Blood levels of epinephrine were significantly increased in castrated mice (Fig. 3A), with no further increase after immobilization stress. Analysis of downstream targets of epinephrine signaling showed comparable increases in phosphorylation of the PKA substrates CREB and BAD in prostates of castrated mice that were or were not subjected to immobilization stress, compared to intact mice (Fig. 3 B,C).

### Surgical Stress Delays Androgen Ablation-induced Prostate Involution

**Figure 4:**
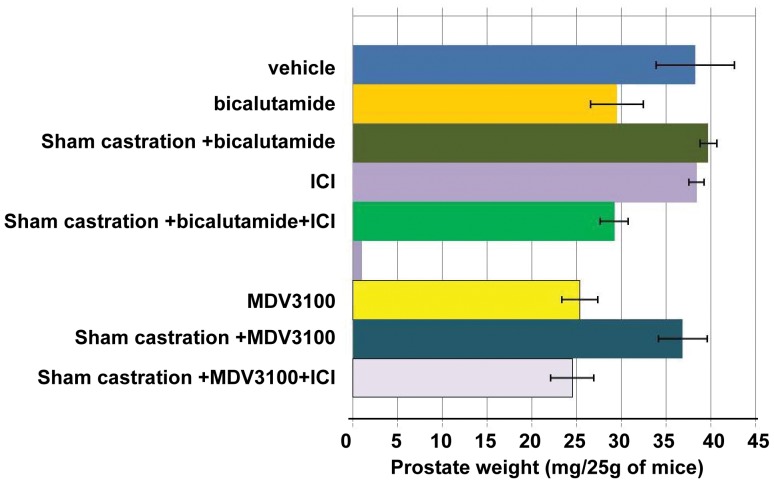
Surgical stress delays prostate involution induced by androgen ablation. C575BL/6J mice received subcutaneous injections of bicalutamide (50 mg/kg, once daily) and MDV3100 (10 mg/kg, once daily) with or without sham castration. ICI118,551 (ICI) was given 30 minutes before sham castration. 2 days later prostates were excised, dissected, and weighted. At least 3 mice were used for each data point. Error bars represent SD from the average.

To test the effects of surgical stress on prostate involution, we compared prostate weights in mice subjected to sham operation (incision of scrotum) or left intact. To induce prostate involution, mice were treated with a clinically used androgen receptor antagonist bicalutamide [11], [12] or MDV3100, a new anti-androgenic drug with higher affinity to the androgen receptor than bicalutamide [13]. Prostates were analyzed on the second day of bicalutamide or MDV3100 treatment, because strongest effect of stress was observed at this time point (Fig. 2A). As shown in Fig. 4, sham castration inhibited prostate involution induced by either bicalutamide or MDV3100. Effects of surgical stress on prostate involution were blocked by ADRB2 antagonist ICI118,551. These results support the hypothesis that surgical stress inhibits apoptosis similar to immobilization stress, and delays involution of prostate gland via epinephrine/ADRB2 signaling.

## Discussion

Normal prostate epithelial and androgen-dependent prostate cancer cells undergo apoptosis when androgen levels are decreased [14]. Evidently androgen is an essential survival factor for androgen-dependent prostate cells [15]. Androgen ablation induced by anti-androgen therapy with bicalutamide or MDV3100 administration in mice can cause significant prostate involution and apoptosis (Fig. 2 and Fig. 4). The AR antagonist MDV3100 is a more potent inhibitor of AR function than bicalutamide because in addition to competing with androgens it prevents AR nuclear localization and DNA binding [13], [16]. Contrary to pharmacological androgen ablation that reduced prostate weight by 29–34% on day 2 and 47% on day 3, surgical androgen ablation by bilateral castration in mice induced delayed prostate involution with no decrease of prostate weight on day 2, 29% on day 3, and 34% on day 4 (Fig. 1, 2, 4). These differences in the dynamics of prostate involution lead us to propose that surgical stress can delay androgen ablation-induced mice prostate involution. Indeed, subjecting mice to sham operation significantly delayed prostate involution on days 2 and 3.

Surgery is known to induce tumor growth [7], [8], [17], [18], and different mechanisms for this effect have been reported, including shedding of tumor cells, vascular endothelial growth factor (VEGF)-induced tumor growth [6], and release of other growth factors and cytokines [19]. These earlier studies implicated surgical stress and activation of the hypothalamic-pituitary-adrenal (HPA) axis in the effects of surgeries on tumor growth, but identified inhibited cell-mediated immunity or stimulated neoangiogenesis as the mechanisms behind these effects [6], [8].

Here we report for the first time that surgical stress can delay prostate involution induced by androgen ablation in mice. Effects of surgical stress were blocked by the selective β2-adrenoreceptor antagonist ICI118,551 and in BAD3SA knockin mice, implicating the β2 adrenergic receptor and BAD phosphorylation as mechanisms for its effect. Surgical castration showed similar effects to immobilization stress by increasing both blood epinephrine levels and CREB and BAD phosphorylation, and delaying apoptosis and prostate involution in mice (Fig. 1 and Fig. 3). Behavioral stress showed no additional effects, suggesting that both surgical and immobilization stress influence prostate involution via the same mechanisms. Thus, the experiments presented here identify direct inhibition of apoptosis in prostate epithelial cells as the primary mechanism by which surgical stress delays prostate involution induced by androgen ablation. These results are consistent with earlier studies showing accelerated tumor growth and inhibition of apoptosis by agonists of β-adrenoreceptors in mouse models of prostate cancer [9], [20]. Future studies using primary and metastatic prostate cancer models will determine whether surgical stress impedes inhibition of prostate tumors by androgen ablation as well as by other prostate cancer therapies.

Results from this study elucidate the mechanisms by which surgical stress can prevent apoptosis in prostate glands. These results justify future epidemiological studies that address possible connections between surgery and prostate cancer progression. Considering the protracted nature of androgen ablation therapy prostate cancer patients may require surgeries to treat complications of prostate cancer or for unrelated reasons [3]–[5]. If a connection between surgical stress and prostate cancer progression is confirmed, preoperative inhibition of β-adrenergic signaling can improve efficacies of androgen ablation therapies.
